# How geoarchaeology and landscape archaeology contribute to niche construction theory (NCT)

**DOI:** 10.1007/s12685-015-0144-8

**Published:** 2015-09-21

**Authors:** Sjoerd J. Kluiving

**Affiliations:** Faculty of Humanities, VU University Amsterdam, De Boelelaan 1105, Room 14A-00, 1081 HV Amsterdam, The Netherlands; Faculty of Earth & Life Sciences, VU University Amsterdam, De Boelelaan 1085, Room O-449, 1081 HV Amsterdam, The Netherlands; Research Institute for the Heritage and History of the Cultural Landscape and Urban Environment (CLUE), De Boelelaan 1105, 1081 HV Amsterdam, The Netherlands

**Keywords:** Geoarchaeology, Landscape archaeology, Landscape gradients, Niche-construction theory (NCT), Anthropocene

## Abstract

In this paper a review is given of examples of geoarchaeological and landscape archaeological research from four locations throughout Europe. Case-studies from the North Sea coastal zone in the Netherlands and the Eastern Mediterranean are presented to illustrate the potential contribution of geoarchaeology and landscape archaeology to niche construction theory (NCT) studies. Typical landscapes as coast lines, lake shores and rivers as example of small and large scale use of the natural landscape and/or topography are discussed with implications for NCT, mainly over the Holocene period. Through environmental reconstruction, we provide relative dates for starting points when humans (a) were altering their own selective environment as an inceptive change, or (b) responded to a (deteriorated) selective environment in a counteractive change. Geoarchaeology and landscape archaeology valuable contribution to NCT studies is the focus of the disciplines on landscape gradients. NCT phase transitions from inceptive to counteractive changes are proposed as useful alternative in the debate about the onset of the Anthropocene.

## Introduction

**Fig. 1:**
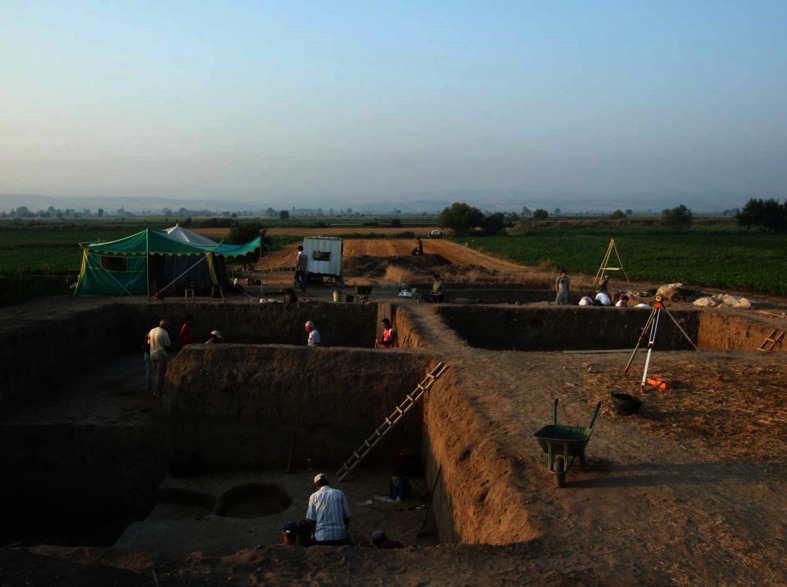
The Neolithic mound of Barcin Höyuk, western Turkey: a seven meter high elevation of cultural layers for more than 7000 years (*photo* F. Gerritsen)

In Western-Turkey, Barcin Hoyük is a 8500 year old Neolithic mound (6500 cal BC) which has been (geo-) archaeologically explored over the past years (*3*. in Fig. 3; Gerritsen et al. 2013a, b; Groenhuijzen et al. 2015). It is the oldest Neolithic site in the region, making it of particular interest regarding the spread of farming from the regions of origin (southeast and central Anatolia) to northwest Anatolia (Fig. 1). The Neolithic settlers used a natural elevation to found their first dwelling place in a generally wet marshy environment, situated on the margin of a retreating lake and likely in the vicinity of a natural stream (Groenhuijzen et al. 2015). A position at the junction of a river or stream and a lacustrine water body demonstrates the strategic importance of a multiple land–water gradient (land–lake and land–river).

This example suggests that a gradient driven selection procedure was used by the first settlers. In such a selection procedure humans favour locations that are located on land–water divides—‘landscape gradients’. On the long term these gradients have a fundamental and irreversible impact on the environment. In this case the start of the mound building phases in the Neolithic have continued into historic (Byzantine) times. For the micro-environment of Barcin Höyuk it can be postulated that since 8500 years ago an unintended change of the landscape has taken place where the first settlers and subsequent cultures have altered their own selective environment completely. The subsequent elevated altitude of the mound by adding cultural layers through time have progressively altered the landscape. It can further be postulated that the ecosystem dynamics in the direct vicinity within and around the mound feature at Barcin Höyuk have been out of ‘natural’ equilibrium starting since 8500 years ago, and may have reached a novel status in the micro-environment that is adapted to local changed circumstances, such as groundwater variations, variable solar input, soil biological variations, resulting in different content of vegetation cover. We consider the mound as the starting point where humans have altered their own selective environment as (a) an inceptive change for the regional context, as well as (b) a counteractive change in the micro-environment where the mound is situated, i.e. exactly valid for the confined site where the cultural layers have been mounted.

**Fig. 2:**
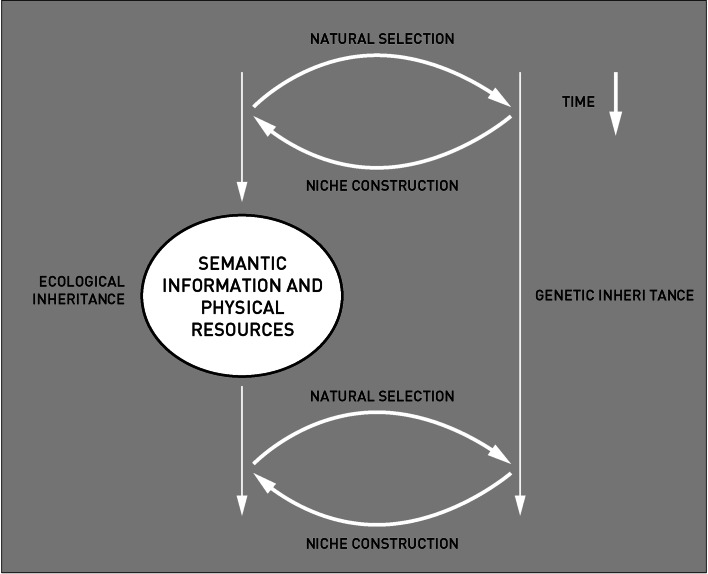
The relations between natural selection and niche construction through time showing two different outcomes, the genetic and ecological inheritance. In the latter process human interference through social learning and physical elements will add an ecological inheritance to the next cycle of natural selection and niche construction (after Kendal et al. 2012)

Over the last 100,000 years, and most likely also earlier, humans have become increasingly reliant on physical and semantic resources produced by cultural activities of preceding generations (Kendal et al. 2012; Smith 2012). It can be argued that such inventions have in various ways depended on and then subsequently shaped the evolution of genetic and other cultural traits. Niche construction theory is the paradigm for studying relationships that recognize the reciprocal influences of cultural evolution, cultural evolvability and gene-cultural evolution (Kendal et al. 2012). In standard evolutionary theory (SET) environmental change depends exclusively on environmental states, evolutionary change in organisms relates to both organisms’ states and environmental states, while adaptive evolutionary change is assumed to be governed by natural selection alone (Fig. 2). In NCT evolutionary change in organisms is similar, but the environmental change is now dependent on both the environmental states and the niche constructing behaviour of organisms (Kendal et al. 2011). Usually and for many instances SET is valid, but it is insufficient when genetic selective environments are modified by processes like the transmittal of cultural knowledge (learning). NCT therefore implies that, caused by previous niche construction, the next cycle not only inherits a genetic signature but also an ecological inheritance (Kendal et al. 2011). The ecological inheritance can be comprised in a form of behaviour (semantic information), through social learning, and by material culture, the so called physical resources of inheritance (e.g. artefacts) (Fig. 2).

The defining characteristic of niche construction is not the modification of environments per se, but rather organism-induced changes in selection pressures in environments. Two types of human niche construction have been distinguished so far. In counteractive change, the next niche construction cycle ‘wholly or partly reverses or neutralizes some prior change in one or more natural selection pressures in their environments’. In inceptive change, the next niche construction cycle ‘introduces a new change in one or more natural selection pressures’ (Odling-Smee et al. 2003). Geoarchaeology has been previously defined as a growing and evolving research discipline at the intersection between geomorphology, environmental history and archaeology (Butzer 2008). Geoarchaeology as a research field continues to grow as more analyses and techniques more typically used in earth and environmental sciences are shown to help interpreting the archaeological record (Diskin et al. 2013). According to Engel and Brückner (2014) geoarchaeology is ‘the science that studies geo-bio-archives in an archaeological context by also considering historical and archaeological data sources in its syntheses’, and they emphasize its multidisciplinary role, as a sub discipline of geomorphology, between the geosciences and cultural sciences. Geoarchaeology especially provides insights into landscape reconstruction, human behaviour, and cultural processes that are a backdrop to landscape change (Kluiving et al. 2015).

Although geoarchaeology considers the systematic integration of earth sciences and archaeology as its objective, major gaps in interdisciplinary knowledge worldwide arise due to the natural tendency for archaeologists and geologists to discuss their own data rather than dealing with the integration of both data records (e.g. Goldberg and Macphail 2006; Holdaway et al. 2010). In addition to the development of geoarchaeology, which is mainly practised by geologists and environmental scientists, landscape archaeology, originally coined as a term to define cultural landscape archaeology (e.g. David and Thomas, 2008), has in the last decade progressed to be the platform of interdisciplinary landscape research where natural and cultural scientists aim to bridge an alpha/beta dichotomy (Kluiving et al. 2012; Kluiving and Guttmann 2012; Bebermeier et al. 2012, 2013). A new approach discusses (theoretical) shifts within landscape archaeology, and explores possibilities for bridging the gaps between environmental and cultural approaches to landscapes and the roles of natural processes and social values in the (trans)formation of landscapes (Kluiving and Guttmann 2012).

In this paper landscape archaeological and geoarchaeological research case studies have been selected in order to test whether HNC cycles can be discerned as well as to establish which criteria can make that distinction. Can we identify shortcomings in NCT that can potentially be solved by concepts or methodologies from geoarchaeology and landscape archaeology? In addition our research aim is to discuss these case studies in NCT terminology, to explore their specific environments of human occupation in terms of ‘landscape gradients’. Important landscape gradients that affect human activity are transitions between land and water, high and low altitudes, different soil properties, salt- versus freshwater environments etc. It is hypothesized that the modification of environmental gradients is an important condition for organism-induced changes in selection procedures.

**Fig. 3:**
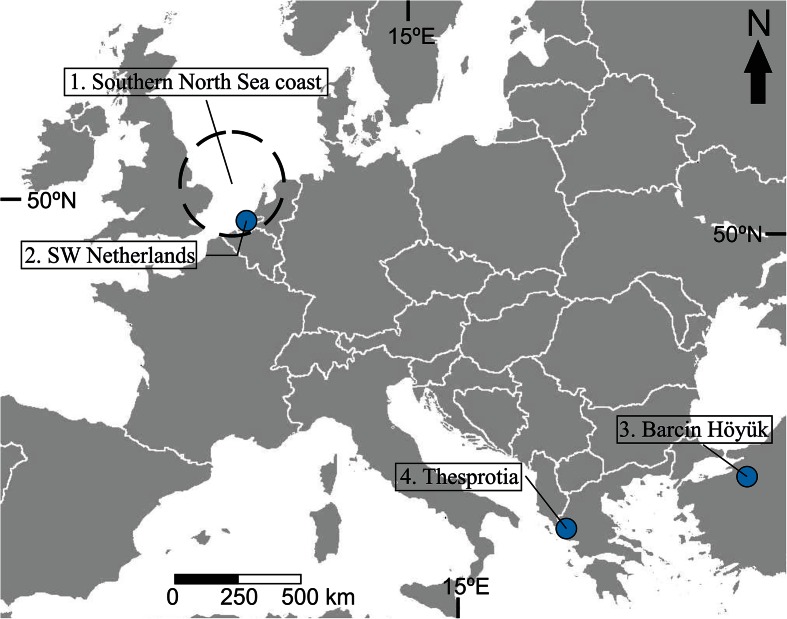
Map of Europe with locations of four geoarchaeological and landscape archaeological case studies presented in this paper

In order to test this hypothesis, it is important to establish criteria to identify NCT in the selected case-studies—e.g. as opposed to a “natural” environment with no human impact or as opposed to the previous cycle of niche construction. Especially in landscapes that typically have spatial and/or temporal gradients, the transition between natural and human-dominated environments might be diffuse. The Netherlands, North Sea Region, as well as a case studies from the Mediterranean area (Greece) are presented to examine and illustrate the potential of geoarchaeology and landscape archaeology for NCT (Fig. 3). Variable scales of landscape research are considered, and the importance of landscape gradients for NCT will be addressed. Coast lines, lake shores and rivers as examples of small and large scale use of the natural landscape and/or topography are considered in the case studies.

## Southern North Sea coast, NW Europe

The next example is derived from human responses to a dynamic landscape and Holocene sea level change (Kluiving et al. 2013; Borger et al. 2011). Originally humans in the coastal parts of the Low Countries faced sea level rise, and responded in a reactive way to the problem of rising water. Due to sea level rise, coastlines in the Netherlands have moved consistently eastward since the end of the last ice age, the Weichselian, and onwards from the onset of the Holocene epoch (Berendsen 2011; de Mulder et al. 2003). Subsidence of the land surface, and water derived from the melting and retreating ice sheet were responsible for relative sea level rise. About 10,000 years ago people already were battling against rising water (Fitch et al. 2005). Mesolithic hunters and gatherers fled from the quickly drowned area invaded by the North Sea. The oldest known archaeological features reactive to rising water is derived from Bergschenhoek, in the central Netherlands, and date from 6200 years BP. At this place the surface was elevated with reed bushes by the inhabitants to stabilise living conditions (Van der Waals 1977). Most likely particular Mesolithic cultures were bound to relative short time periods where sand barriers (early coastal barriers) or elevated river beds offered dry conditions.

Relative sea level rise in combination with basin bathymetry and soil properties led to different subsistence strategies in variable tidal inlets in the central Netherlands. If after sea level rise or stream channel diversion the once dry locations were flooded, people moved away or adapted to this new situation, e.g. promoting the adoption of crop cultivation caused by the increased extent of wetland area (Van de Biggelaar et al. 2014). In terms of coastal management it can be stated that people at that time were reactive (in a passive mode) against the threat of the rising water (Fig. 3). The reed bushes or choice for (temporary) higher or dryer places constitute an inceptive change in NCT terminology. Around 5000 years BP, when the glacial ice sheets were finally melted, only subsidence due to regional tectonics remained which resulted in a slower rising sea level. From that time on, the coast started to secure itself by building up a series of elongated sand barriers that shielded the inland from coastal erosion processes, so that humans from that time had increased natural shelter against relative sea level rise. Coastal zones became increasingly inhabited, which left an imprint on the landscape. In addition the relative sea level rise in the last 3000 years in the southern part of the North Sea cannot be solely explained by geological factors (Kluiving et al. 2013). Already 2000 years ago human influence had become a dominant factor in steering natural processes in the coastal zone (De Gans 2007; Vos and Van Heeringen 1997; de Ridder 1999, Fig. 4).

**Fig. 4:**
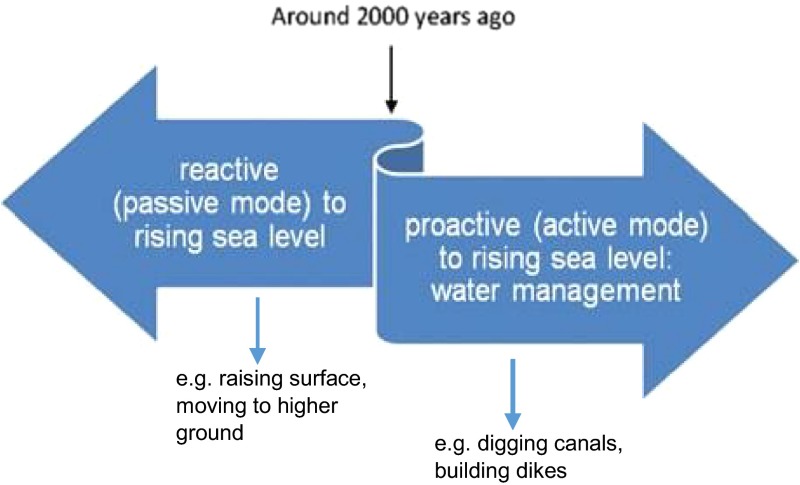
Schematic diagram shows change in reaction of humans against rising sea level and coastal hazards throughout the Holocene in the Netherlands (adapted from Borger et al. 2011)

After the introduction of agriculture, forest clearance took off at a continental scale, creating around 3000 BP large open spaces and heathland in a wide area (De Gans 2007). Indirectly, forest clearance leads through increased run-off and river bed load to a change in coastal morphology, like e.g. delta or spit formation (James and Lecce 2013). Around 2000 years ago humans changed their attitude against coastal defence: instead of just reacting to higher water levels by raising surface level in a passive mode they changed to a proactive way of water management during Roman times and even earlier such as the construction of dams, dikes, dwelling mounds (the first since 2500–2600 BP) and canals (De Gans 2007; De Ridder 1999, Fig. 4). The effects of deforestation on river sedimentation processes and early water management measures can be considered as (proactive) counteractive changes in their respective niche cycle, as opposed to the earlier reactive mode that constituted inceptive changes of niche formation in the coastal landscape.

## SW Netherlands

In the south-western Netherlands specific historical features of a vanished and drowned landscape in the western part of the Dutch province of North-Brabant testify to an almost self-induced disaster that struck inhabitants of several Late Medieval villages (Kluiving et al. 2006; de Kraker and Kluiving 2010). In the southern part of this area, Pleistocene sandy soils are dominant on a relatively higher elevation that is gently sloping down towards the north and (more steeply to the) west, where mainly clayey soils and remnants of peat occur, lying slightly above sea level. On both soil types agriculture was the main kind of land use, although parts of the sandy soils were also wooded. There used to be large scale peat quarrying as well. On the higher sandy part this was mainly for fuel, whereas in the lower areas peat was also quarried for salt extraction.

The history of the area over the last 1000 years is characterized by two settlement periods, a medieval one and a modern one. The end of the medieval settlement period was caused by flooding of the northern lowlands which penetrated eastwards in a series of discrete storm events within a 175 year time span. A number of 40 medieval sites were drowned in this small region of West-Brabant, compared to more than 100 drowned villages in the western province of Zeeland (Kluiving et al. 2006; Kuiper 2004). On a local scale, inhabitants of the medieval village Hildernisse were apparently not able or willing to prevent damage control of the dikes. This may have indirectly caused flooding of the village (Kluiving et al. 2006). On this local scale, such a reactive response, or rather a non-reactive response to the threat of sea level rise, led to a counteractive change. On a regional scale, the exhaustion and industrial use of the peat layers has led to an unprecedented surface lowering and an irreversible human-induced change (that led to significant drowning of the inhabited landscape). Exploitation of the widely available peat landscape combined with oxidation and compaction through drainage has caused a significant lowering of the natural surface up to approximately 10 m in the entire coastal zone of NW Europe (Berendsen 2011).

Although re-embankment already started at the same time, the final and largest scale flooding occurred during the 80 year’s War (1568–1648), which also hit the western lowlands. This long term flooding affected the lowlands of the landscape tremendously. Large parts of the flooded landscape were eventually covered with a thick layer of clay that also covered most medieval settlements and large parts of the old infrastructure, freezing it in time. In other parts flooding was destructive, because new channels were formed that completely washed away all archaeological remnants (i.e. counteractive changes).

Thus, in the SW Netherlands, a medieval drowned landscape was created by nature and humans (Kluiving et al. 2006). Planned and unplanned behaviour of ‘natural’ systems, coupled with the unintended consequences of inhabitants, resulted in a complete ‘make-over’ of the landscape. Widespread flooding caused or augmented by human interference was an unintentional change of the environment and as such an unintentional change of the accompanying ecosystems. This process relates closely to concepts of NCT, with humans inducing modification in an environment of counteractive changes.

**Fig. 5:**
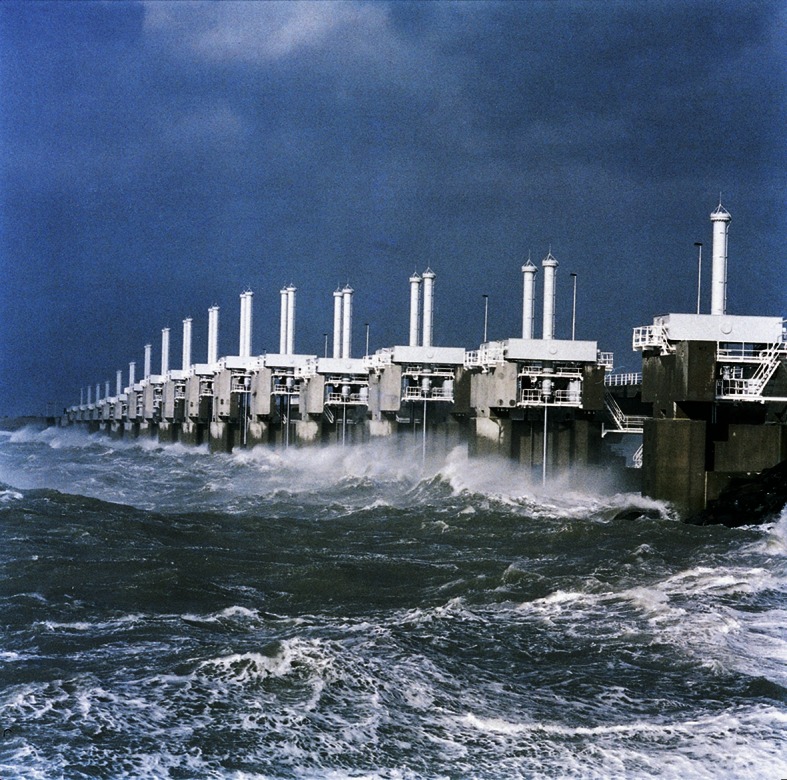
Storm surge barrier in the ‘Oosterschelde’ estuary, signature of coastal defence after 1953 disaster that flooded the south-western part of the Netherlands (Zeeland;* photo* A.M.J. de Kraker)

After the sixteenth and seventeenth century, the area has seen a process of widespread land reclamation and dike building, with the result that eighteenth, nineteenth and early twentieth century inhabitants of SW Netherlands have felt relatively safe from flooding. Unusual natural forces, i.e. combinations of storm levels and wind directions, co-created the big flooding disaster in February 1953 that flooded vast areas. 72.000 people were evacuated and 1836 people drowned (Borger et al. 2011). During the second half of the twentieth century, the erection of mega-structures for coastal defence in the South-Western Netherlands is a large-scale human-induced landscape change. Until now, these structures are the ultimate pro-active response of humans to sea level rise and coastal defence issues (Fig. 5; Borger et al. 2011).

## Thesprotia, North-western Greece

Within the western part of Greece, in the Epirus region, and subregion of Thesprotia several (former) lake environments have been studied in geoarchaeological projects to unravel environmental conditions with human presence throughout the Quaternary period (Kluiving et al. 2011; Lelivelt 2011). In Lake Limnoula several cycles of rising and falling of the water level can be related to climatic oscillations of wettening and drying. The sediments of the lake are highly bioturbated and disturbed, due to the soil formation processes (Kluiving et al. 2011). These pedogenetic processes, though destructive for the palaeobotanical reconstruction study, can be used as chronostratigraphic evidence of climatic stability and stable landscape conditions. Combined with radiocarbon dating, they can be correlated to archaeological periods in nearby valleys (e.g. Kokytos valley, Thesprotia).

In Lake Kalodiki the pollen sequence suggests a possible early Weichselian dating of lake sediments, whereas the same sedimentological sequence continues until the late Weichselian (Kluiving et al. 2011). This period can be related to the Middle to Upper Palaeolithic archaeological framework and is represented by rich scattered finds and sites in the wider environment of Epirus. In the proximity of Lake Kalodiki, Palaeolithic tools at the site of Morphi are relatively dated to the Middle and Upper Palaeolithic period. At least during the Middle Palaeolithic period the basin of Kalodiki was enclosed and the surrounding hills were covered with dense vegetation of Quercus. Kalodiki formed one of the numerous basins (poljes) that are found in Epirus, which would have provided a favourable environment to the Palaeolithic populations, constituting a refugee for animals and plant species and providing a secure source of water within an inhospitable mountainous environment (Kluiving et al. 2011). Lake Kalodiki is an example of a land–water gradient which attracts humans to settle, comparable to other land–water gradients. The presumed influence of Middle to Upper Palaeolithic inhabitants to their environment at Kalodiki will have been an inceptive change in the HNC cycle. The assumption is that Middle to Upper Palaeolithic inhabitants changed natural selection pressures by their presence in the landscape, e.g. by the use of fire, or hunting wildlife.

**Fig. 6:**
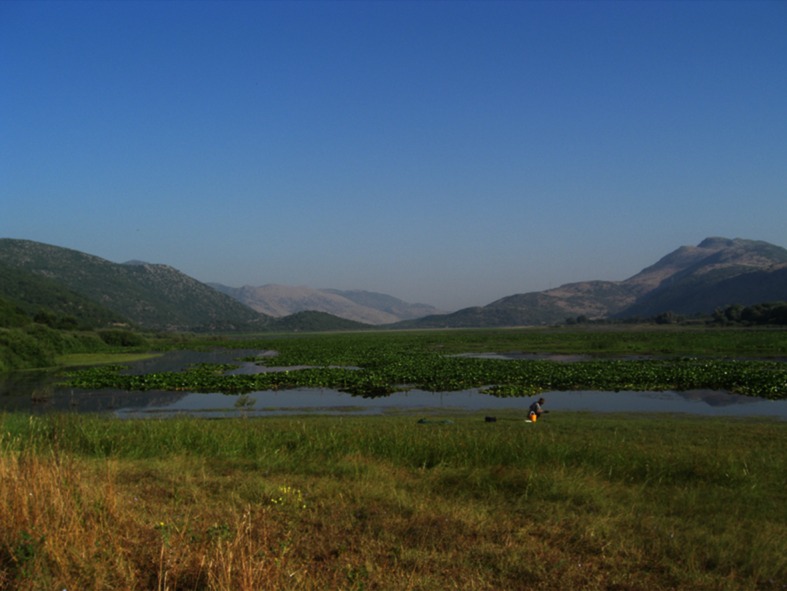
Southern lake shore of Lake Kalodiki where cored sediments show evidence of deforestation and increased runoff in the past. The increased runoff has most likely resulted in the deposition of slope fans that have reached the lake shore as is shown in the picture (*photo* R. Lelivelt 2011)

Deforestation caused by human impact will change the sediment budget and affect natural processes like river meandering, or initiate slope processes that in turn may affect human presence in the landscape. For Lake Kalodiki, geoarchaeological research has concluded that human impact between 3250 and 2000 cal. BC has influenced the sedimentary pattern in the lake basin (Lelivelt 2011). The lithostratigraphical profiles of cored sediments demonstrated changing sedimentary environments within this time period (Fig. 6). Slope processes are suspected to have initiated when humans removed the vegetation cover that influences the stability of the soil against slope gravity processes. The removal of trees and vegetation is a human-induced process and, since the impact on natural processes is paramount, is in this respect an example of a counteractive change. It can be argued that the infilling of the lake is a result of soil erosion upstream and inland caused by deforestation. The infilling then has the unintended consequence of humans creating a modified environment which is then taken advantage of by the inhabitants of the lake basins. The reconstructed Holocene processes with slope failure through deforestation differ from the stable landscape situation during Palaeolithic habitation for which no such changes have been recorded.

## Discussion

In cases where humans have changed natural processes beyond the intrinsic natural periodicity of a process—like sea level rise—we argue that the large-scale culturally induced natural system fundamentally alters ecosystems as well. It is actually a challenge to understand the dynamics of ecosystems in the absence of human pressure (Donlan et al. 2006). We observe that humans take actions and measures, such as deforestation, widespread peat exploitation, and water management, without necessarily knowing which consequences their activities have on natural processes, like relative sea level rise, river sedimentation, or slope stability. The balance between nature and culture are under continuous discussion (e.g. Zalasiewicz et al. 2010, 2015; Kaplan et al. 2009; Ruddiman, 2013; Smith and Zeder 2013; Foley et al. 2013), including the debate about the onset (and meaning) of the Anthropocene. This debate has so far received little incorporation of archaeologists (Erlandson and Braje 2013). We propose a further integration of methodologies and concepts in the broad field of human-environment interaction, and propose an NCT concept in order to extend the current discussion about the Anthropocene (cf. Smith and Zeder 2013).

Our set of geoarchaeological examples of human-environment interaction demonstrate that natural processes in various European regions over different time transects are intrinsically influenced by cultural evolutions (Table 1). Evidence from landscapes from older civilizations like the Palaeolithic cultures in Epirus, Western Greece, show an almost undisturbed ecosystem. Most proxies testify of a natural sequence of events like wet and dry cycles, as well as stable soil formation conditions, the latter offering in general a potential for Niche Construction on a regional scale. The Palaeolithic example in Thesprotia suggests human impact, based on hunting and burning behaviour in the lake shore environment, as an inceptive change (Table 1: 4a). The Holocene example from Thesprotia shows an unintentional human-induced slope processes due to forest removal on a local scale. As such the land-lacustrine landscape gradient is the starting point where man partly reverses a prior change in one or more natural selection pressures in the lacustrine environments (counteractive change; Table 1: 4b). The Neolithic mound of Barcin Höyuk is an example of a Human Niche constructed on a local scale at land-lacustrine gradient with a counteractive change, while the regional surroundings had less effect and show an inceptive change (Table 1: 3).

**Table 1:** Considering the examples of geoarchaeological research in this paper we can list them with respect to location, landscape gradient type, archaeological period, geological period process, scale and NCT mode of change

Nr.	Location	Landscape gradient type	Archaeological period	Geological period	Process	Scale	NCT mode of change
1a	Southern North Sea coast I	Land-sea	Mesolithic	Early-Mid Holocene	Relative sea level rise	National	Inceptive
1b	Southern North Sea coast II	Land-sea	Bronze-Iron Age	Mid-Late Holocene	Deforestation, water management	National	Counteractive
2a	SW Netherlands	Land-sea	Late-Medieval, New age	Late Holocene	Peat erosion, relative sea level rise	Regional	Counteractive
2b	SW Netherlands	Land-sea	Late-Medieval, New age	Late Holocene	Peat erosion, relative sea level rise	Local	Counteractive
2c	SW Netherlands	Land-sea	New Age	Late Holocene	Relative sea level rise, severe storms, coastal defence	Regional	Counteractive
3a	Barcin, W. Turkey	Land-lacustrine Land-river	Neolithic-present	Holocene	Lake level fluctuations, ecosystem dynamics	Regional	Inceptive
3b	Barcin, W. Turkey	Land-river	Neolithic-present	Holocene	Lake level fluctuations, ecosystem dynamics	Local	Counteractive
4a	Thesprotia, Greece I	Land-lacustrine	Palaeolithic	Pleistocene	Lake level fluctuations, ecosystem dynamics	Regional	Inceptive
4b	Thesprotia, Greece II	Land-lacustrine	Bronze-Iron Age	Mid-Holocene	Deforestation, land slides	Local	Counteractive

North Sea coastal dynamics have shaped the southern North Sea coast during the early and mid-Holocene. On a regional scale transformations occur when humans are at first reacting to rising water in a passive mode (inceptive change), but change to proactive water management (Table 1: 1). In the latter case, it can be discussed further if and how changes in water management structures as well as governmental steering have led to HNC (Wilkinson et al. 2012). The fact that humans had a dominant role in steering natural processes since the last 3000 years has a profound impact on how we see intentional and unintentional effects. The best example of an unintentional effect on a regional scale is the drowned landscape in the south-western part of the Netherlands. The medieval drowned landscape shows evidence of a human induced modification of the environment according to the principles of NCT in a counteractive change (Table 1: 2b). In the twentieth century, governmental steering led to the erection of one of the greatest coastal defence systems in the world as a counteractive change in the NCT cycle (Table 1: 2c; Fig. 5).

The observations from the case study areas (Table 1) suggest a transition in NCT mode throughout the Holocene period. In the Early to Mid-Holocene human impact can be modelled as inceptive changes in the NCT cycle, such as reactive modes to relative sea level rise. Early pre-Holocene hunter-foragers in Thesprotia influenced ecosystems only locally, which we have labelled as inceptive changes. However all changes in the Mid- to Late Holocene period can be interpreted as counteractive, involving deforestation, dam building, peat exploitation with usually irreversible, long-lasting and large scale events and processes. Niche construction has also been labelled as ecosystem engineering and serves as such as the ‘cause’ for the effects we see in the environmental records (Smith and Zeder 2013). Humans have the ability to create new niche constructing behaviours and to transpose these behaviours through social learning. This reinforced capacity to modify environments up to a societies standards makes humans the ‘ultimate niche constructors’ (Odling-Smee et al. 2003; Smith 2007, 2012). While Smith and Zeder (2013) consider the initial domestication of plants and animals worldwide as coeval with the Holocene epoch, we propose an alternative in analysing changes in human’s impact on ecosystems and landscapes, considering the mode of change, i.e. inceptive or counteractive. An inceptive change is usually the first unintentional attempt to introduce a new change in the natural selection procedures (cf. Odling-Smee et al. 2003). Every first encounter with human impact of a natural ecosystem will go through this first change of NCT. Continued human impact time after time will eventually lead to a reversal or neutralisation of selection pressures in ecosystems that can be labelled as counteractive changes.

Evaluating the two-part division in HNC modes (Odling-Smee et al. 2003) through our cases, we propose that the inceptive/counteractive tipping point in ecosystems, although regionally diachronous, might be a consistent marker for the Holocene-Anthropocene boundary. Although reconstruction of past states of ecosystems is challenging (Donlan et al. 2006), geoarchaeological and landscape archaeological research can assist in unravelling different modes of NCT, and therefore contribute to the discussion about the onset of the Anthropocene. The systematic use of NCT terminology embedded in geoarchaeological research will enable another objective criterion for discussing the onset of the Anthropocene. Our cases show two distinct phases at the transition from societies adapting to prevailing conditions towards societies altering their niche. Increased time control at geoarchaeological study sites will enable studying when these important transitions took place. Landscape gradients—linear elements (lake shore, coasts line) in a landscape—are vulnerable for unintended impacts when settled by humans. Early inceptive changes would be followed by counteractive changes, when human impact has left a substantial mark in the landscape. As such, the human niche construction mode is time dependent. The Barcin example suggests that there is an additional scale dependency: very small, micro-environments may show counteractive changes, while larger regional scales show inceptive changes at the same time (Table 1). Geoarchaeology and landscape archaeology can be of value for NCT in answering these questions and analysing this chronological and spatial dependency of human niche cycles in relation to landscape gradients.

 We call for a multi-disciplinary approach in HNT studies in which geoarchaeology has a key role to demonstrate the nature-culture dichotomy in order to make important contributions to Human Niche Construction Theory (e.g. Van den Biggelaar and Kluiving 2015). By illuminating the concept of landscape gradients as determining for settlement and niche constructions geoarchaeology can analyse parameters of the ‘pure’ unchanged natural environment right before the period in time humans had their influence. As such the discipline can deliver ‘baseline’ data of the pre-human landscape in the discussion of HNC in order to determine which type of landscape was preferred at what time by ancient civilisations. This approach will make a contribution to the application of NCT in the Anthropocene debate (cf. Smith and Zeder 2013).

## Concluding remarks

The observation of the shift from reactive to proactive water management styles through time in the western Netherlands around 3000 years ago runs parallel with the recent discussion on the Anthropocene, which places the onset of dominant human influence on nature in the Late Iron Age (Kaplan et al. 2011). However a number of other researchers place the start of the Anthropocene much earlier in the Holocene (Ruddiman 2013; Ruddiman et al. 2008; Smith and Zeder 2013). An alternative discussion dealing with the start of the Anthropocene ranges from the nineteenth century Industrial Revolution age to the launching of the first nuclear atom bomb during the mid-twentieth century (Zalasiewicz et al. 2010, 2015). Supporting the late Anthropocene onset, the ‘Palaeoanthropocene’, i.e. the entire period with humans present preceding the Industrial Revolution, is proposed as a period of small and regional effects of human-environment interaction (Foley et al. 2013). NCT is a powerful concept that encompasses an integration of research disciplines, such as geology and archaeology, in dealing with these issues. Incorporating NCT in the Anthropocene discussion contributes to the debate about cause and effect of human-environmental interaction, and will call upon (geo-)archaeological syntheses to confirm or reject transitions from societies adapting to prevailing conditions towards societies altering their niche. We call for the International Commission on Stratigraphy, the Anthropocene Working Group of the Subcommission on Quaternary Stratigraphy, to acknowledge the start of an interdisciplinary scientific debate about one of the most significant time periods in the Earth’s history, and to postpone a formal decision on the onset of the Anthropocene, in order to stimulate future research themes.
